# Phage-driven coevolution reveals trade-off between antibiotic and phage resistance in *Salmonella anatum*

**DOI:** 10.1093/ismeco/ycae039

**Published:** 2024-03-22

**Authors:** Yuanyang Zhao, Mei Shu, Ling Zhang, Chan Zhong, Ningbo Liao, Guoping Wu

**Affiliations:** College of Animal Science and Technology, Jiangxi Agricultural University, Nanchang 330045, Jiangxi, PR China; College of Food Science and Engineering, Jiangxi Agricultural University, Nanchang 330045, Jiangxi, PR China; College of Food Science and Engineering, Jiangxi Agricultural University, Nanchang 330045, Jiangxi, PR China; College of Animal Science and Technology, Jiangxi Agricultural University, Nanchang 330045, Jiangxi, PR China; College of Food Science and Engineering, Jiangxi Agricultural University, Nanchang 330045, Jiangxi, PR China; College of Food Science and Engineering, Jiangxi Agricultural University, Nanchang 330045, Jiangxi, PR China; College of Food Science and Engineering, Jiangxi Agricultural University, Nanchang 330045, Jiangxi, PR China

**Keywords:** Salmonella anatum, phage, coevolution, trade-off, phage resistance, gene mutation

## Abstract

Phage therapy faces challenges against multidrug-resistant (MDR) *Salmonella* due to rapid phage-resistant mutant emergence. Understanding the intricate interplay between antibiotics and phages is essential for shaping *Salmonella* evolution and advancing phage therapy. In this study, MDR *Salmonella anatum* (*S. anatum*) 2089b coevolved with phage JNwz02 for 30 passages (60 days), then the effect of coevolution on the trade-off between phage and antibiotic resistance in bacteria was investigated. Our results demonstrated antagonistic coevolution between bacteria and phages, transitioning from arms race dynamics (ARD) to fluctuating selection dynamics (FSD). The fitness cost of phage resistance, manifested as reduced competitiveness, was observed. Bacteria evolved phage resistance while simultaneously regaining sensitivity to amoxicillin, ampicillin, and gentamicin, influenced by phage selection pressure and bacterial competitiveness. Moreover, the impact of phage selection pressure on the trade-off between antibiotic and phage resistance was more pronounced in the ARD stage than in the FSD stage. Whole genome analysis revealed mutations in the *btuB* gene in evolved *S*. *anatum* strains, with a notably higher mutation frequency in the ARD stage compared to the FSD stage. Subsequent knockout experiments confirmed BtuB as a receptor for phage JNwz02, and the deletion of *btuB* resulted in reduced bacterial competitiveness. Additionally, the mutations identified in the phage-resistant strains were linked to multiple single nucleotide polymorphisms (SNPs) associated with membrane components. This correlation implies a potential role of these SNPs in reinstating antibiotic susceptibility. These findings significantly advance our understanding of phage-host interactions and the impact of bacterial adaptations on antibiotic resistance.

## Introduction

The use of antibiotics in medicine and agriculture has led to the emergence of multidrug-resistant (MDR) bacterial pathogens [1], which are predicted to cause 10 million deaths per year by 2050 [2]. Virulent bacteriophage (phage) has been proposed as a potential therapeutic agent for recalcitrant bacterial infections [3]. However, bacteria can develop resistance to phages through a variety of mechanisms, such as horizontal gene transfer (HGT) [4] and mutations of outer membrane receptors [5], which correspondingly provide an opportunity for adapted phages to exploit resistant bacteria. This antagonistic interaction between phages and bacteria drives changes in the trait distribution of populations [6], with bacterial resistance to phage evolution generally dominating the coevolutionary system [7]. Therefore, the efficacy of phage therapy may be compromised if phages are unable to effectively reduce bacterial density, often attributable to bacteria evolving phage resistance [8]. Evolutionary theory predicts that resistance to phages should be associated with a fitness cost, as the lack of a cost would result in the emergence and maintenance of all pathogens as resistant [9]. To counter this, one approach is to capitalize on evolutionary trade-offs between phage resistance and either antibiotic sensitivity [10] or reduced bacterial virulence [11–13]. For instance, research has identified a reduction in efflux pump efficiency associated with phage resistance, which can lead to a decrease in antibiotic resistance [3, 14, 15]. In particular, the phages OMKO1 in *Pseudomonas aeruginosa* and TLS in *Escherichia coli* have been found to cause resistance loss to antibiotics [16]. Thus, exploiting the evolutionary trade-offs between phage resistance and antibiotic sensitivity can be a promising therapeutic strategy [17].

The antagonistic coevolution of hosts and their parasites generally has two models: (i) arms race dynamics (ARD) driven by directional selection, which favors a wider range of resistance in the host against a great number of parasite genotypes and a greater host range in the parasites, allowing for more host genotypes to be infected [18, 19]; and (ii) fluctuating selection dynamics (FSD), which is characterized by a lack of directional change in the evolution of the host’s resistance range, is governed by negative frequency-dependent selection, favoring hosts that resist the most commonly encountered parasite genotypes and parasites that infect the most common host genotypes [19–21]. ARD is a typical short-term coevolutionary pattern that decelerates over time. The major reason is the increasing fitness costs of generalism for phages and bacteria with increasing infectivity and resistance. In contrast, when costly resistance mutations are no longer beneficial to bacteria, it may lead to long-term coexistence between bacteria phages by FSD coevolution [22]. The effects of the two coevolutionary dynamics on the trade-off between phage and antibiotic resistance in bacteria may be different. For example, Kortright *et al.* [23] found that in three repeated coevolution experiments of *P. aeruginosa* and phage OMKO1 communities, ARD occurred in one of the experiments, whereas the other two showed evidence for FSD. Furthermore, only the ARD bacteria showed a trade-off between phage and antibiotic resistance. The factors affecting coevolution are complex, and the competitiveness of bacteria for resources may be an important factor. Studies have shown that bacterial resistance to phages is often associated with reduced competitiveness for resources [24, 25]. This may be due to the fact that more phages use other membrane components rather than drug efflux pumps as receptors, such as lipopolysaccharides and TonB-dependent Vitamin B12 receptor (BtuB) [26], which directly affect bacterial competitiveness but may indirectly mediate the trade-off between phage and antibiotic resistance. Additionally, limited resources in specific coevolution systems may also be a contributing factor to this trade-off [27]. In general, little is known about how coevolution may affect the trade-off of phage resistance and antibiotic resistance in bacteria. It will be important to determine the changes in coevolutionary dynamics during the coevolution and whether or not this could impact the trade-off between phage resistance and antibiotic resistance of bacterial populations under phage selection pressure.

*Salmonella* is a common food-borne pathogen that can cause serious public health threats [28], and *S. anatum* is one of the important serotypes of *Salmonella* spp., which is widely prevalent in Asia [29]. The growing prevalence of antibiotic-resistant *Salmonella* strains has led to an increased recognition of phages as novel bactericidal agents for controlling *Salmonella*, especially MDR strains. It is critical to understand the influence of phage parasitism on *Salmonella* population biology and antibiotic resistance, given the organism’s ubiquity in natural habitats, as well as its widespread presence in food and hospitals [30]. Previous studies have shown that both empirical and theoretical models of *Salmonella enteritidis* and its virulent phage show a mutational asymmetry dynamic in favor of the bacteria instead of antagonistic coevolution [31, 32]. However, there is still a limited understanding of the interactions between selection from antibiotics and phages and their role in driving *Salmonella* evolution, due to the complexity of the environment, phage diversity, and bacterial hosts involved in these interactions [5, 33].

**Figure 1:**
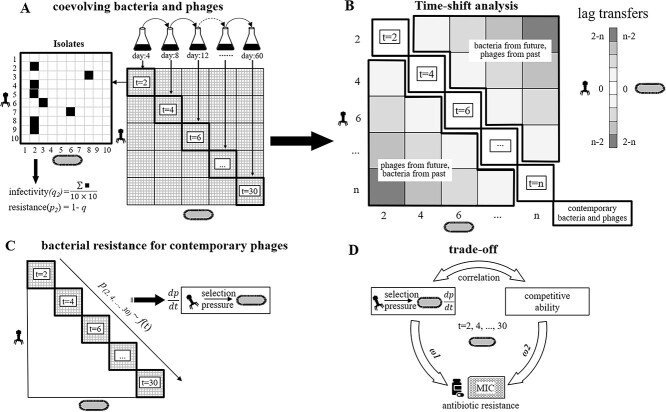
The experimental design for studying the coevolution between *S. Anatum* 2089b and its virulent phage JNwz02; (A) the coevolution of bacteria and phages was performed through batch co-culture with serial transfer; 10 phages and bacteria were isolated every second transfer (4 days) from the culture; the infectivity/resistance in every phage/bacteria combination was measured by the spot testing, and a cross-infection matrix was obtained; four replicates were conducted, and a control without phage addition was performed in parallel; (B) a time-shift assay was used to determine if bacteria and phages co-evolved during the experiment; the cross-infection were classified into three groups: (i) contemporary bacteria and phages (black box, both phages and bacteria were isolated from transfer *t*); (ii) phages isolated from future and bacteria isolated from past (green box, phages isolated from transfer *t* + 2, bacteria isolated from transfer *t*); and (iii) phages isolated from past and bacteria isolated from future (blue box, phages isolated from transfer *t* − 2, bacteria isolated from transfer *t*); (C) the changes of bacterial resistance to contemporary phages over time was fitted as a model and derivative of the fitted model ($\frac{dq}{dt}$) was calculated to quantify the effects of phages on bacterial resistance (i.e. phage selection pressure); (D) trade-off between antibiotic resistance and phage resistance; the relative importance of phage selection pressure (*ω*_1_) and bacterial competitiveness (*ω*_2_) on antibiotic resistance during the coevolutionary experiment.

In this study, we aimed to investigate the effect of phage selection pressure on the evolution of antibiotic resistance in *S. anatum*. To achieve this goal, we established experimental microcosms and allowed *S. anatum* 2089b, a MDR strain isolated from raw pork, to interact and potentially coevolve with its virulent phage JNwz02 over a 60-day period consisting of 30 serial transfers (see Fig. 1 for details). We then assessed the competitiveness associated with bacterial resistance to phage infection and determined the level of antibiotic resistance in the coevolved bacteria. We also performed whole-genome sequencing and analysis of phage-resistant mutants obtained at different transfer times to elucidate the mutations that arose in response to phage selection pressure. Our study will offer a deep insight into the dynamics of phage-driven antibiotic resistance in *S. anatum*, thereby presenting significant implications for future endeavors aimed at enhancing the efficacy of antimicrobial treatments.

## Materials and methods

### Bacterial strains and phages

To initiate coevolution between bacteria and phages, *S. anatum* strain 2089b (GenBank accession number: CP118633.1) and its virulent phage JNwz02 (GenBank accession number: MZ570151.1) were employed. Our previous studies [28] identified *S. anatum* 2089b isolated from food as MDR (Table 1). The phage JNwz02, isolated from domestic sewage and displaying high lytic activity against the host bacterial strain *S. anatum* 2089b [28], was employed as the predator. Furthermore, a gift of the *E. coli* O157:H7 9940 strain from Prof. Robert E. Levin (Department of Food Science, University of Massachusetts, USA) was utilized for the competition assay.

**Table 1:** Details of the 10 antibiotics and the antibiotic resistance of the *S. anatum* 2089b strain.

**Number**	**Antibiotic**	**Types of antibiotics**	**MIC of the WT 2089b (μg/ml)**
1	Sulfamethoxazole	Sulfonamides	2000
2	Ciprofloxacin	Quinolones	50
3	Nalidixic acid	Quinolones	1000
4	Tetracycline	Tetracyclines	250
5	Florfenicol	Chloramphenicol	250
6	Neomycin	Aminoglycosides	31.25
7	Gentamycin	Aminoglycosides	125
8	Amoxicillin	β-lactams	500
9	Ampicillin	β-lactams	2000
10	Cefotaxime	β-lactams (cephalosporin)	2

### Batch co-culture with serial transfer

The batch co-culture experiment was conducted following the protocol laid out by Osada *et al.* [34], with some minor adjustments. At the start, 10 ml of tryptic soy broth [TSB; Becton, Dickinson and Company (BD), Sparks, MD, USA] in a 50-ml shake-flask was inoculated with 100 μl of an overnight culture of wild-type (WT) 2089b at 37 °C, using a shaker incubator set to 200 rpm. After 1.5 h of incubation, the bacterial culture (OD600nm ≈ 0.5) had reached a density of ~10^8^ CFU/ml and was then infected with JNwz02 at a titer of 10^6^ PFU/ml, resulting in a multiplicity of infection (MOI) of 0.01. The bacteria and phage were then allowed to coevolve for a 48-h period at 37 °C with shaking at 200 rpm, after which 1% (100 μl) of the culture was transferred into 10 ml of fresh TSB broth. This procedure was repeated 30 times (spanning a total of 60 days) by serial transfer. Four replicates were conducted, and a control without phage addition was performed in parallel, under the same conditions.

Every second transfer (every 4 days, i.e., 15 times for the entire coevolutionary process; Fig. 1A), 600 μl of culture was frozen at −80 °C in a 20% v/v glycerol-TSB solution. One milliliter of the culture was centrifuged at 12 000 g for 5 min at 4 °C; the pellet was then washed four times with sterile physiological saline (0.85% NaCl) to remove free phages. Dilutions of 100 μl were plated onto tryptic soy agar (TSA; BD Diagnostic Systems, Sparks, MD, USA), and, after 16–18 h of incubation at 37 °C, the number of colonies was counted. Phages were isolated by adding 100 μl of chloroform to 900 μl of the culture, then centrifuging at 12 000 g for 15 min to lyse and pellet bacterial debris. The phage titer of the supernatant was determined by serial dilution with sterile SM buffer (10 mM MgSO_4_, 100 mM NaCl, 0.01% gelatin, 50 mM Tris-HCl, pH 7.5), followed by plaque assay on lawns of WT 2089b.

### Isolation of coevolved bacteria and phages

To assess the alterations in infectivity and resistance over time, 10 phage plaques and bacterial colonies (clonal isolates) were isolated from the culture after every second transfer. The bacteria in the culture of every second transfer were diluted and plated onto tryptic soy agar (TSA). Then, 10 single colonies were randomly picked from the plate and inoculated into 3 ml tryptic soy broth (TSB) and cultured for 16–18 h at 37 °C with shaking at 200 rpm, respectively. Finally, 600 μl of each single colony culture was stored in 20% glycerol at −80 °C for subsequent analysis. To detect potential contamination in our co-culture systems, the colors and morphology of the bacterial colonies formed on TSA plates were visually compared with those of WT 2089b. In the event of any suspected contamination, partial sequences of the 16S rRNA genes of the bacterium were determined by PCR using 341F and 907R primers [34].

The phages from every second transfer were diluted with SM buffer and followed by plaque assay on lawns of WT 2089b, 10 individual plaques formed on a WT 2089b lawn were individually collected and amplified in 3 ml of TSB broth with the WT 2089b strain as host. Following 16–18 h of incubation at 37 °C with shaking at 200 rpm, 1.5 ml of the culture was added with 10% v/v of chloroform, and the mixture was vortexed and centrifuged at 12 000 g for 15 min at 4 °C to separate the phages. The supernatant containing phages was filtered with a 0.22 μm pore size filter, and then 600 μl of the supernatant was frozen at −80 °C in 20% glycerol, and the remaining supernatant was stored at 4 °C for subsequent analysis. This approach to phage isolation may disadvantage phage clones that are unable to grow on WT bacteria, although a previous study revealed such phage phenotypes to be rare [22]. This procedure yielded a total of 150 host and 150 phage clonal isolates for the batch co-culture experiment (15 time points × 10 isolates = 150 isolates).

### Infectivity and resistance assays

The infectivity/resistance in every phage/bacteria combination was measured by spot testing (150 phages × 150 bacteria = 22 500 assays, Fig. 1A). For each assay, the lawn was poured with 100 μl of 10^8^ CFU/ml host bacteria (10^7^ cells) in a soft TSB agar plate with an inner diameter of 85 mm. The density of bacteria on the lawn was about 1.8 × 10^3^ cells/mm^2^. Then, 2 μl of phage lysate at a titer of 10^8^ PFU/ml was added to a lawn of the relevant host bacteria and incubated at 37 °C for 16–18 h. Phage suspension of 2 μl formed a circle with a diameter of about 4 mm, and the density of phages was about 1.6 × 10^4^ particles/mm^2^. Therefore, the MOI was about 9. Phages were scored as infective if the spotted area was observed to be a clearing zone. Infectivity (*p*)/resistance (*q*) at each combination of bacteria time (*n*, i.e. the transfer that bacteria were isolated from) and phage time (*m*, i.e. the transfer that phages were isolated from) was calculated as the proportion of successful infections/resistances by the following equations [35]:


(1) \begin{equation*} {p}_{nm}=\frac{\sum \mathrm{number}\ \mathrm{of}\ \mathrm{visible}\ \mathrm{plaque}}{10\ {\mathrm{bacteria}}_n\times 10\ {\mathrm{phages}}_m} \end{equation*}

(2) \begin{equation*} {q}_{nm}=1-{p}_{nm} \qquad\qquad\qquad\qquad\ \qquad\end{equation*}

where *n* and *m* denote the transfer that bacteria and phages were isolated from, respectively, and the range of infections and resistances is 0 ≤ *p_nm_*, *q_nm_* ≤ 1.

### Time-shift assays: detecting coevolution between bacteria and phages

Based on the spot test described above, a time-shift assay approach described by Betts *et al.* [35] was used to determine if bacteria and phages evolved adaptations and counter-adaptations antagonistically. The pairwise interactions (i.e. phages isolated from transfer *m* infecting host bacteria isolated from transfer *n*) during the batch co-culture experiment were classified into three groups (Fig. 1B): (i) interactions among contemporary bacteria and phages (i.e. both phages and bacteria were isolated from transfer *t*); (ii) interactions among phages isolated from future points in time and bacteria isolated from past points in time (e.g. phages isolated from transfer *t* + 2, bacteria isolated from transfer *t*); (iii) interactions among phages isolated from past points in time and bacteria isolated from future points in time (e.g. phages isolated from transfer *t* − 2, bacteria isolated from transfer *t*). A monotonous increase in resistance/infectivity from the past to the present to the future is considered as the signature of an escalating coevolutionary arms race. Nonlinear trajectories, in particular local maxima or minima of resistance/infectivity, indicate fluctuating coevolutionary dynamics, which can be driven by FSD (e.g. Red Queen dynamics [36]).

### Fitness cost of resistance assays

To determine whether bacterial resistance to phages was associated with a fitness cost (competitiveness) in the absence of phages, a competition assay was performed as described by Hall *et al.* [22] with some modifications. In brief, the competitiveness of the WT 2089b and 150 evolved host bacteria was determined by a competition assay against an *E. coli* O157:H7 9940 strain. For each assay, the host bacteria (WT 2089b or evolved host bacteria) and *E. coli* O157:H7 were incubated to the mid-log phase of growth (~10^8^ CFU/ml), respectively. Hence, both competitors were in comparable physiological states. Approximately 10^7^ cells from each competitor were then added to 10 ml of fresh TSB broth and incubated for 24 h at 37 °C with shaking at 200 rpm. Densities of both competitors at the start and end of the assay were estimated by plating onto eosin methylene blue (EMB) agar; on these plates, the *E. coli* O157:H7 strain forms fluorescent green colonies, whereas *Salmonella* produces white colonies. Competitiveness was taken as the ratio of the estimated Malthusian parameters (*m*) of each competing type, *m* = ln(*N_f_*/*N*_0_), where *N_0_* is the initial and *N_f_* is the final density [22]. The relative competitiveness (*W*) of the evolved host bacteria relative to WT 2089b was calculated by the following equation:


(3) \begin{equation*} {W}_{ni}=\frac{m_{ni}}{m_{\mathrm{WT}}} \end{equation*}

where *n* denotes the transfer that bacteria was isolated from, and *i* denotes each evolved host bacteria from transfer *n*. The mean relative competitiveness (*W_n_*) of 10 isolated bacterial strains from transfer *n* was calculated by the following equation:


(4) \begin{equation*} {W}_n=\frac{\sum_{i=1}^{10}{W}_{ni}}{10}. \end{equation*}

When *W_n_* < 1, *W_n_* = 1, and *W_n_* > 1, it indicates that the mean fitness of host bacteria from transfer *n* has decreased, not changed, or increased compared with WT 2089b, respectively.

### Antibiotic resistance levels assays

Minimum inhibitory concentration (MIC) assays were determined by a 2-fold dilution technique in 96-well microtiter plates, as described by the Clinical and Laboratory Standards Institute (CLSI) guidelines. The antibiotic resistance level of the WT 2089b and 150 evolved host bacteria was measured as the MIC at which no bacterial growth was detected. For each strain, the original solutions of 10 antibiotics (ampicillin, amoxicillin, cefotaxime, tetracycline, gentamycin, neomycin, florfenicol, ciprofloxacin, nalidixic acid, and sulfamethoxazole) were diluted to the test concentrations at 2-fold decrements in Mueller–Hinton (MH) broth, respectively. Bacterial culture was diluted to ~2 × 10^5^ CFU/ml in MH. MICs for each antibiotic were measured by dispensing 100 μl of a given antibiotic concentration and 100 μl of diluted bacteria into wells of a 96-well plate and incubating at 37 °C with shaking at 200 rpm for 12 h. Bacterial growth was scored visually as a binary variable (no growth vs. any visible growth). The lowest antibiotic concentration at which the well was completely clear was the value recorded as the MIC. For each antibiotic, the relative antibiotic resistance (*R*) of the evolved host bacteria relative to WT 2089b was calculated by the following equation:


(5) \begin{equation*} {R}_{ni}=\frac{{\mathrm{MIC}}_{ni}}{{\mathrm{MIC}}_{\mathrm{WT}}} \end{equation*}

where *n* denotes the transfer that bacteria was isolated from, and *i* denotes each evolved host bacteria from transfer *n*. For each antibiotic, the mean relative antibiotic resistance (*R_n_*) of 10 isolated bacterial strains from transfer *n* was calculated by the following equation:


(6) \begin{equation*} {R}_n=\frac{\sum_{i=1}^{10}{R}_{ni}}{10}. \end{equation*}

When *R_n_* < 1, *R_n_* = 1, and *R_n_* > 1, it indicates that the mean antibiotic resistance of host bacteria from transfer *n* has decreased, not changed, or increased compared with WT 2089b, respectively.

### Adsorption rate assays

The bacterial cells of 10^8^ CFU/ml were mixed with JNwz02 at an MOI of 0.001. The adsorption between bacteria and phages was performed for 15 min at 37 °C. Then, the bacterial cells were centrifuged at 12 000 g for 5 min at 4 °C. The phage titer of the supernatant was determined by serial dilution with sterile SM buffer, followed by a plaque assay on lawns of 2089b. The phage adsorption rate was calculated by the following equation [37]:


(7) \begin{align*} &\mathrm{Adsorption}\ \mathrm{rate}\ \left(\%\right)\nonumber\\&=\frac{\mathrm{initial}\ \mathrm{phage}\ \mathrm{titer}-\mathrm{phage}\ \mathrm{titer}\ \mathrm{in}\ \mathrm{the}\ \mathrm{supernatant}\ }{\mathrm{initial}\ \mathrm{phage}\ \mathrm{titer}}\times 100\%. \end{align*}

### Knockout of *btuB* gene

For the *btuB* knockout assay, a *Salmonella* strain 1093b, one of the hosts of JNwz02 [28], was used. The deletion mutant of *btuB* in the *Salmonella* strain was constructed using the λ-red recombination system as previously described [26]. Briefly, the FRT-flanked kanamycin resistance gene cassette from pKD4 was PCR amplified using appropriate primers and transformed into a *Salmonella* strain containing pKD46. The primers used were: *btuB*-F (5′-GATTAAA AAAGCTTCGCTGCTGACGGCGTGTTCCGTCACGGTGTAGGCTGGAGCTGCTTC-3′) and *btuB*-R (5′-ACCGACGCCGGAGTCAAACACCAGCACGGTGGGAC GTGGTCATATGAATATCCTCCTTAG-3′). Each primer contained the target gene sequence, shown underlined. After selection in the presence of kanamycin and ampicillin, pKD46 was cured as described by Hong *et al.* [26]. The genome-integrated kanamycin resistance gene was then removed by the introduction of pCP20, followed by the curing of pCP20.

### Bacterial genome resequencing and bioinformatic analysis

The genomes of 10 isolated *S. anatum* strains isolated from transfers 4, 6, 8, 10, 12, 14, 16, 20, 24, and 30 were sequenced for all four coevolutionary replicates. Whole genome sequencing of the strains isolated from transfers 8, 14, 20, 26, and 30 in the control was also performed. The genomic DNA of bacteria was extracted and purified according to the previously described method [38]. The genomes were sequenced using Illumina Novaseq 6000 (Illumina, San Diego, CA, USA), and the raw data were filtered using Trimmomatic v0.39 [39]. The filtered sequencing data were aligned to the reference genome by using BWA v0.7.17 [40] and SAMtools v1.7 [41]. The mutant sites of the evolved bacteria were analyzed by using SAMtools v1.7, BreakDancer v1.4.5 [42], and VarScan v2.4.4 [43]. The genetic polymorphisms were determined based on the minor allele frequency, which was computed by VarScan v2.4.4. Gene ontology (GO) (http://geneontology.org/) enrichment analysis was conducted on genes with polymorphism sites in the genomes of bacterial strains.

### Statistical analysis

The evolution of phage infectivity and bacterial resistance during the batch co-culture experiment was investigated by employing a general linear model (GLM) [quasi-binomial error distribution and link function = logit] to analyze the cross-infectivity data.

To analyze the changes in phage resistance with transfer times during the coevolutionary experiment, the GLM was used to model the probability of phage resistance with a quasi-binomial distribution (link function = logit). The transfer time was fitted as a continuous variable with linear and nonlinear (quadratic, cubic, and quartic) polynomial terms. The derivative of the fitted model was calculated to quantify the effects of phages on bacterial resistance during the coevolutionary experiment (i.e. the change rate of bacterial resistance over transfer time $\frac{dq}{dt}$). When $\frac{dq}{dt}$ > 0 or $\frac{dq}{dt}$ < 0, it indicates that phages steer bacterial resistance to increase or decrease at transfer *t*, respectively; and when $\frac{dq}{dt}$ = 0, it indicates that phages have no effect on bacterial resistance at transfer *t*.

Competition experiments (values of *W*) were analyzed as a linear model (LM) with transfer time (*t*) fitted as a variable. To determine if there was an absolute cost of resistance relative to the WT 2089b, one-sample *t*-tests of the relative competitiveness were carried out against the control. The trade-off between the bacterial resistance (*q*) to phages and the relative competitiveness (*W*) was assessed using a LM. Bacterial resistance data in the above analyses were square-root arcsine transformed to meet model assumptions.

To analyze the changes in antibiotic resistance with transfer times during the coevolutionary experiment, the GLM was used to model the probability of antibiotic resistance with a quasi-binomial distribution (link function = logit). The transfer time was fitted as a continuous variable, and the treatment (experimental treatment with phages vs. control treatment without phages) was fitted as a factor.

To analyze the effect of phage resistance and relative competitiveness on antibiotic resistance, the LM was fitted with antibiotic resistance as the response variable. The phage resistance and the relative competitiveness (and their interaction) were fitted as continuous variables. To assess the extent of the effect of phage selection pressure and relative competitiveness on antibiotic resistance at the ARD stage and FSD stage, respectively, the relative importance of the variables was calculated using the relimplm function (R packages “relaimpo”), respectively. The Chi-squared test was used to determine whether the relative importance of the two variables was significantly different between the two stages. All statistical analysis was conducted in R version 4.0.5 (R Core Team 2021, https://www.R-project.org/).

## Results

### Long-term coexistence of *S. anatum* and its virulent phage

**Figure 2:**
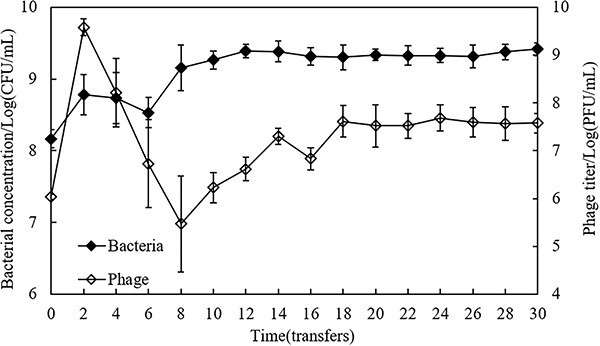
Bacterial concentration (closed diamonds) and phage titer (open diamonds) over time during batch co-culture. The error bars represent the standard deviation of four replicates.

Bacterial concentration and phage titer from batch co-culture samples are shown in Fig. 2. In the initial stages of the serial transfer (transfer 2–8), phage titer plummeted significantly from 9.58 ± 0.17 to 5.47 ± 1.00 log_10_ PFU/ml. Subsequently, in the later stages of the serial transfer (transfer 10–30), an augmentation in phage titer and a subsequent stabilization were observed. For bacteria, in the initial stages of the serial transfer (transfer 2–6), bacterial concentration slightly declined from 8.78 ± 0.28 to 8.53 ± 0.21 log_10_ CFU/ml. Compared with the initial stages, bacterial concentration increased significantly (*t*_13_ = −12.20, *P* < 0.01) and remained in an apparent stationary phase in the later stages (transfer 8–30). In contrast, the bacterial concentration in the control sample without phages was sustained at around 10^9^ CFU/ml without significant alteration. The bacteria concentration and phage titer were ultimately stabilized at a constant level (~10^9^ and 10^7^ PFU/ml, respectively) during the serial transfer, indicating the establishment of a stable coexistence of phages with bacterial populations.

### Coevolutionary dynamics during the batch co-culture of *S. anatum* and phages

The cross-infection matrices of the bacteria-phage combinations were used to determine the coevolutionary dynamics between *S. anatum* and phages during the batch co-culture experiment. For bacteria, there was an increase trend in phage resistance during the initial stages (transfer 2–10), while the phage resistance was maintained at a stable level during the later stages (transfer 12–30, Fig. S1). This result indicates that the interactive pattern between bacteria and phages may be different over time during the batch co-culture experiment. Conversely, the bacteria in the control sample without phages remained sensitive to the WT phage from transfer 2 to 30 (data not shown).

**Figure 3:**
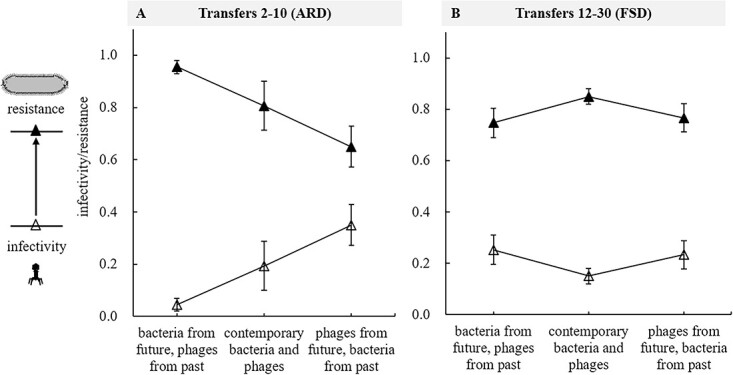
Coevolutionary dynamics for *S. anatum* coevolving with phage JNwz02. Phage infectivity and bacterial resistance (i.e. 1-infectivity) was computed for contemporary bacteria and phages (i.e. both were isolated at the same point in time) and when bacteria (phages) were facing phages (bacteria) either from the past or the future through a coevolutionary experiment. Triangular symbols and error bars represent the mean and confidence interval at 95% of infection and resistance probabilities.

To infer the distinctive feature of coevolutionary dynamics, the pairwise interactions during the batch co-culture experiment were classified into three groups and analyzed according to the methods in “Materials and methods.” The analysis results revealed that the coevolutionary interactions differed significantly (χ2 *df* = 1 = 43.23, *P* < 0.001, Fig. 3 and Table S1) between the initial stages (transfer 2–10) and later stages (transfer 12–30) during the batch co-culture experiment. In addition, bacterial resistance and phage infectivity differed significantly (χ2 *df* = 2 = 256.92, *P* < 0.001) among the three groups during the batch co-culture experiment, i.e. bacterial resistance to past, contemporary, and future phages was significantly different and vice versa for phage infectivity. In the initial stages, bacteria were more resistant to past phages and became less resistant to contemporary and future phages, which is a distinctive feature of ARD (Fig. 3A). Conversely, in the later stages, bacteria were more resistant to their contemporary than past and future phages, which is consistent with FSD (Fig. 3B). The above results suggested that *S. anatum* and phages co-evolved during the batch co-culture experiment and that coevolutionary dynamics was ARD in the initial stages of coevolution and FSD in the later stages.

### Changes in bacterial resistance to contemporary phages during the batch co-culture experiment

**Figure 4:**
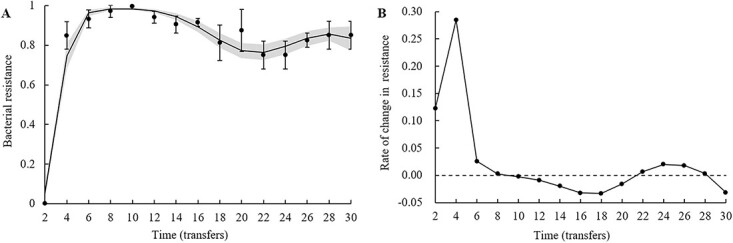
Changes in bacterial resistance to contemporary phages occurred during the batch co-culture experiment. (A) Bacterial resistance to phages over time during a coevolutionary experiment was computed for contemporary bacteria and phages. Solid line represents the means of resistance computed by the generalized linear mode $y=\frac{\exp \left(-0.00017{x}^4+0.014{x}^3-0.40{x}^2+4.23x-10.00\right)}{1+\exp \left(-0.00017{x}^4+0.014{x}^3-0.40{x}^2+4.23x-10.00\right)}$, *R*^2^ = 0.92. Shaded regions are 95% confidence interval of the fitted model. The error bars represent the standard deviation of four replicates. (B) Evolutionary rate of phage resistance was computed from the derivative of the mode. The dotted line indicates that the evolution rate is 0 (i.e. no change in phage resistance). The rate greater than 0 means phage resistance is increasing, otherwise it means phage resistance is decreasing.

The bacterial resistance to phages from these same time points was analyzed (Fig. 4A). Phage resistance increased from 0 to 0.0.9713 over the course from transfers 2 to 8, and reached a maximum of 0.9975 at transfer 10. Then in the later stages (transfer 12–30), phage resistance decreased and subsequently fluctuated in the range of 0.75–0.94 was observed. However, although phage resistance increased to very high levels, phages were always under selective pressure for bacteria, as shown by a decrease in infectivity (Fig. 4A, infectivity = 1 − resistance) from 1 to 0.0025, followed by an increase and fluctuation in the range of 0.059–0.25. To analyze the changes in phage resistance over the course of serial transfer, the generalized LM of phage resistance (*q*) was fitted with transfer time (*t*) as a continuous variable: $q=\frac{\exp \left(-0.00018{t}^4+0.014{t}^3-0.38{t}^2+3.91t-9.33\right)}{1+\exp \left(-0.00018{t}^4+0.014{t}^3-0.38{t}^2+3.91t-9.33\right)}$, *R*^2^ = 0.94 (Fig. 4A). The changes in phage resistance showed nonlinearity, resulting in significant quadratic (*F*_1,12_ = 40.13, *P* < 0.001), cubic (*F*_1,11_ = 64.50, *P* < 0.001), and quartic (*F*_1,10_ = 18.18, *P* = 0.015) polynomial terms of the transfer time variable (Table S2).

To quantify the effects of phages on bacterial resistance to phages during the coevolutionary experiment, the slope ($\frac{dq}{dt}$, i.e. the rate of changes in phage resistance) of phage resistance with respect to transfer time was computed (Fig. 4B). The slope provides a measure of the selective pressure of phages on bacteria over time, with a positive slope indicating an increase in phage resistance (i.e. increased selective pressure on bacteria by phages) and a negative slope indicating a decrease in phage resistance (i.e. decreased selective pressure on bacteria by phages). The slope remained positive over the course from transfer 2 to 8, and was almost zero (−0.0025) at transfer 10. Then in the later stages (from transfer 12 to 30), the slope decreased and subsequently fluctuated in the range of −0.033 − 0.020 was observed. The above results indicated that the stronger selective pressure exerted by phages on bacteria resulted in increasing phage resistance in the initial stages (ARD, from transfer 2 to 10), whereas in the later stages (FSD, from transfer 12 to 30), the selective pressure was weakened, so that phage resistance was maintained at a stable level.

### The trade-off between phage resistance and competitiveness during the batch co-culture experiment

**Figure 5:**
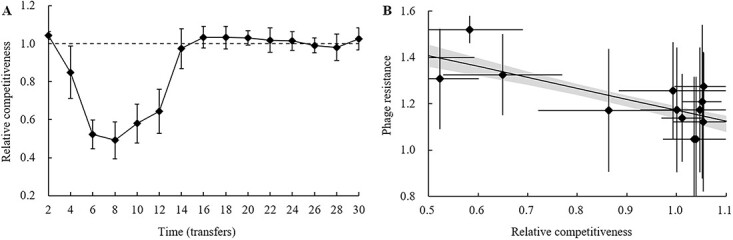
Trade-offs between phage resistance and relative competitiveness during coevolutionary experiment. (A) The changes of competitiveness of bacteria relative to WT 2089b over time during coevolutionary experiment. (B) Trade-offs between phage resistance and relative competitiveness during coevolutionary experiment. Solid line represents the regression equation between phage resistance and fitness cost computed by linear mode: *y* = −0.4986*x* + 1.7194, *R*^2^ = 0.52. Shaded regions are 95% confidence intervals of the fitted model. The error bar represents the mean and standard deviation of four replicates.

The changes in relative competitiveness of bacteria during the coevolutionary experiment are shown in Fig. 5A. Relative competitiveness decreased from 1.04 ± 0.02 to 0.49 ± 0.09 over the course of transfers 2–8, and increased to 0.58 ± 0.10 at transfer 10. Then in the FSD stage (from transfer 12 to 30), relative competitiveness increased and subsequently fluctuated in the range of 0.64–1.03 was observed. The LM of relative competitiveness was fitted with transfer time as a continuous variable (*R*^2^ = 0.91, Fig. 5A and Table S3). The changes in relative competitiveness showed nonlinearity, resulting in significant cubic (*F*_1,11_ = 41.93, *P* < 0.001) and quartic (*F*_1,10_ = 26.93, *P* < 0.001) polynomial terms of the transfer time variable (Table S3). The above results indicated that the bacterial competitiveness decreased significantly relative to the ancestral strain in the ARD stage, whereas in the FSD stage, the competitiveness was restored and maintained at a stable level. In contrast, the bacterial relative competitive ability in the control sample without phages was maintained at ~1.09 without significant change.

The trade-off between the phage resistance and the relative competitiveness was assessed using a LM. Bacteria strains during batch co-culture experiments show a fitness cost (i.e. competitive ability) with increasing bacterial resistance (Fig. 5B; linear regression: *y* = −0.49*x* + 1.65, *R*^2^ = 0.62, *P* < 0.001). The above results indicated that bacterial resistance to phages was associated with a fitness cost during batch co-culture experiment, i.e. the higher the bacterial resistance to phages, the lower their competitiveness. Indeed, in the face of phage predation, the bacterial hosts are often forced into a trade-off between becoming resistance against virulent phage predation and the fitness costs associated with maintaining this resistance [44, 45].

### Changes in bacterial antibiotic resistance during the batch co-culture experiment

**Figure 6:**
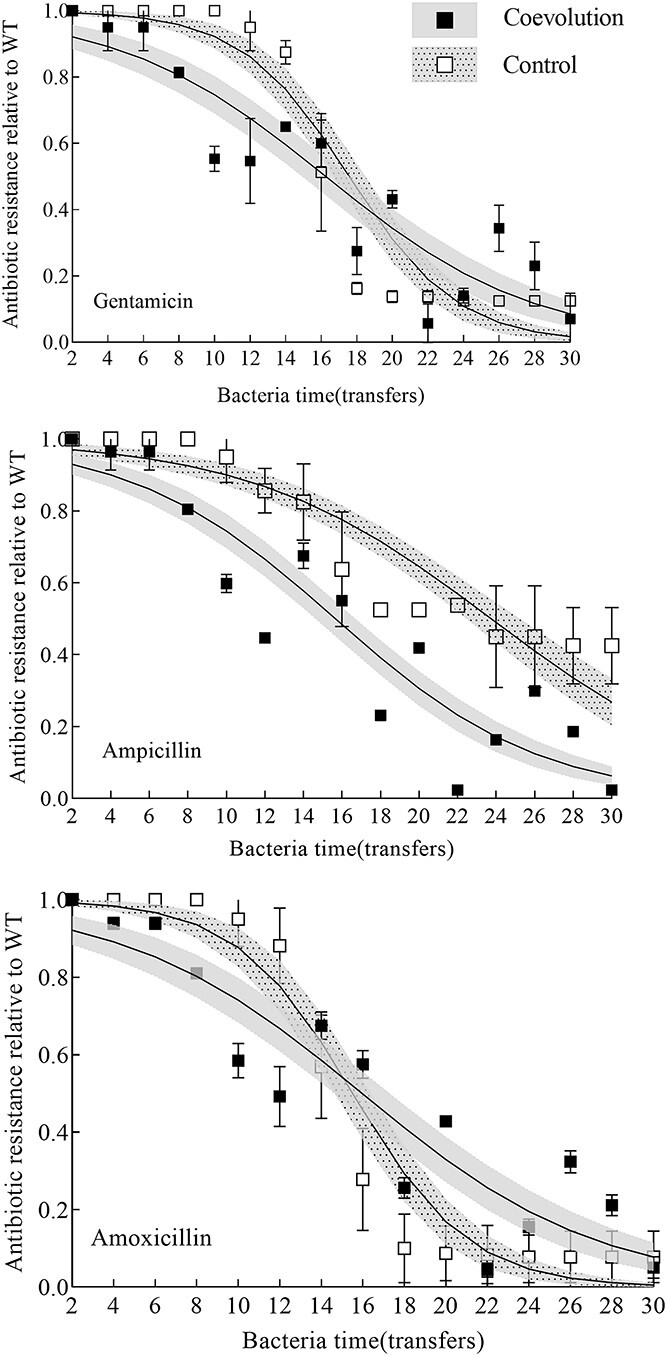
Changes in antibiotic resistance of *S. anatum* over time during the coevolutionary experiment. The antibiotic resistance of bacteria has decreased significantly to amoxicillin (*F*_1,28_ = 56.28, *P* < 0.001), ampicillin (*F*_1,28_ = 103.07, *P* < 0.001), and gentamycin (*F*_1,28_ = 95.41, *P* < 0.001) over time, and there were significant differences compared with the control without phage during the experiment (amoxicillin: χ2 df = 1 = 5.42, *P* = 0.019; ampicillin: χ2 df = 1 = 14.41, *P* < 0.001; gentamycin: χ2 df = 1 = 4.45, *P* = 0.035). The error bar represents the mean and standard deviation of four replicates.

The changes in antibiotic resistance of every transfer of bacteria relative to the WT 2089b strain were expressed as relative antibiotic resistance. Details of the 10 antibiotics and antibiotic resistance of the WT 2089b strain are shown in Table 1. The antibiotic resistance of bacteria has decreased significantly to amoxicillin (*F*_1,28_ = 128.47, *P* < 0.001), ampicillin (*F*_1,28_ = 114.78, *P* < 0.001) and gentamycin (*F*_1,28_ = 15.64, *P* < 0.001) over time, and there were significant differences compared with the control without phage during the experiment (amoxicillin: χ*2 df = 1* = 6.36, *P* = 0.011; ampicillin: χ*2 df = 1* = 22.36, *P* < 0.001; gentamycin: χ*2 df = 1* = 7.64, *P* < 0.001; Fig. 6). In contrast, there was no significant difference in the relative antibiotic resistance of bacteria to the other seven antibiotics over time (Fig. S2 and Table S4). These results indicate that the evolution of bacterial resistance to phages significantly affected the changes in antibiotic resistance to amoxicillin, ampicillin, and gentamycin compared to the control without phages. Therefore, the changes in antibiotic resistance of bacteria to these three antibiotics over time were selected for further analysis.

Bacteria that had been co-cultured with phages showed earlier reductions of antibiotic resistance to all three antibiotics compared to control without phages (Fig. 6). In contrast, the changing trend of antibiotic resistance varied in the later stages of the serial transfer, depending on treatment. While antibiotic resistance of bacteria to amoxicillin and gentamycin was maintained at a stable level from transfer 16 to 30 in control without phage, more fluctuations were observed in the co-cultured treatment with phage (Fig. 6). Together, these results suggest that competition within the bacterial population is also a factor driving bacterial evolution, resulting in reduced antibiotic resistance. Moreover, the antibiotic resistance of bacteria to three antibiotics showed a consistent changing trend over time in the co-cultured treatment with phage, which is reflected by the significant positive correlation among the three antibiotics (*r* > 0.99, *P* < 0.01; Fig. S3). This result suggests that mutations resulting from bacteria responding to phage infection may pleiotropically affect a common node in the three antibiotic resistance mechanisms, such as permeability.

### Trade-off between antibiotic resistance and phage resistance

To determine that changes in antibiotic resistance may be driven by selective pressure of phages, a multiple LM was fitted with antibiotic resistance as the response variable, selective pressure of phages (i.e. the rate of changes in phage resistance), and relative competitiveness were fitted as continuous variables. The results (Table 2) showed that the changes in antibiotic resistance to three antibiotics were significantly correlated with selective pressure of phages and bacterial competitiveness during the coevolutionary experiment (gentamycin: *F*_3,10_ = 10.15, *P* = 0.0022; ampicillin: *F*_3,10_ = 8.76, *P* = 0.0038; amoxicillin: *F*_3,10_ = 6.92, *P* = 0.0084). By considering both variables together, they explained more than 68%–75% of the variance in three antibiotic resistance changes (gentamycin: *R*^2^ = 0.75; ampicillin: *R*^2^ = 0.72; amoxicillin: *R*^2^ = 0.68; Table 2). Thus, the combined effect of phage selection pressure and bacterial competitiveness had the major influence on the antibiotic resistance of bacteria to three antibiotics.

**Table 2:** The significance test of the linear regression analysis model was performed with antibiotic resistance as the response variable and phage selective pressure (i.e. the rate of change of bacterial resistance) and competitiveness were fitted as continuous variables.

**Antibiotic**	**Significance**	**Coefficients**			
		**Source**	**Estimate**	***F***	***P***
Gentamycin	*R* ^2^ = 0.75 *F*_3,10_ = 10.15 *P* = 0.0022	Rate of change in resistance	38.03	8.23	0.017
		Relative competitiveness	−0.88	15.39	0.0029
		Rate × competitiveness	−42.23	6.83	0.026
Ampicillin	*R* ^2^ = 0.72, *F*_3,10_ = 8.76 *P* = 0.0038	Rate of change in resistance	35.39	8.29	0.016
		Relative competitiveness	−0.93	13.47	0.0043
		Rate × competitiveness	−38.89	4.52	0.059
Amoxicillin	*R* ^2^ = 0.68 *F*_3,10_ = 6.92 *P* = 0.0084	Rate of change in resistance	31.47	5.86	0.036
		Relative competitiveness	−0.88	11.47	0.0069
		Rate × competitiveness	−34.68	3.45	0.093

A significant interaction was found between phage selective pressure and bacterial competitiveness on antibiotic resistance evolution (rate × competitiveness, see Table 2), with a significant interaction in gentamycin (*F*_1,10_ = 6.83, *P* = 0.026) and a generally significant interaction in ampicillin (*F*_1,10_ = 4.52, *P* = 0.059) and amoxicillin (*F*_1,10_ = 3.45, *P* = 0.093). These results indicate that the relationship between the antibiotic resistance and one of the variables depends on the level of the other variable. The effect of bacterial competitiveness on antibiotic resistance increased with the increase in phage selective pressure (Fig. S4). Together, these results suggest that phage selection pressure is a major driver of the evolution of bacterial resistance to antibiotics during the coevolutionary experiment. In addition, phages may have pleiotropically driven antibiotic resistance evolution through some indirect pathway, i.e. phages drive evolutionary trade-offs with bacterial competitiveness, which in turn interacts with bacterial competitive ability to affect changes in antibiotic resistance.

### The role of phage selection pressure and bacterial competitiveness in antibiotic resistance at two stages of coevolution

**Figure 7:**
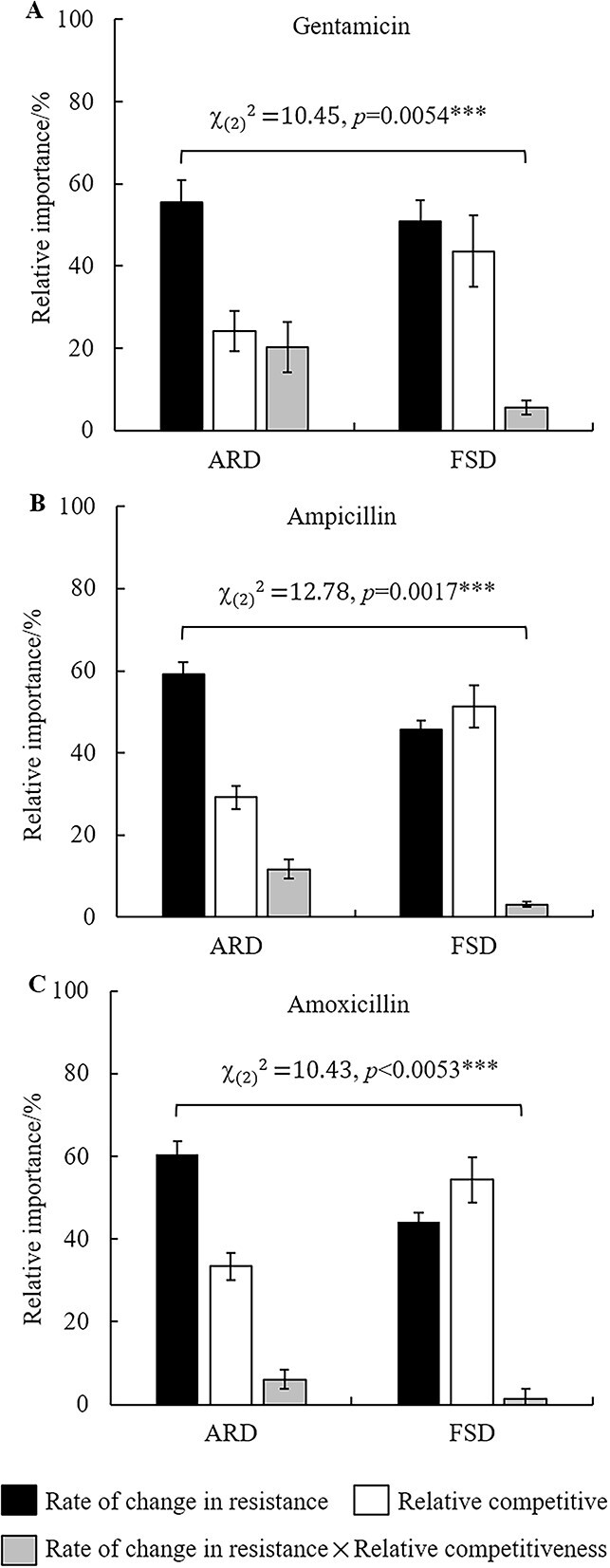
The importance of phage selection pressure and bacterial competitive ability on antibiotic resistance at different stages. The relative importance of phage selection pressure (i.e. rate of change in resistance) and bacterial competitive ability on bacterial resistance against gentamicin (A), ampicillin (B) and amoxicillin (C) was significantly different at two stages (transfer 2–10, ARD; transfer 12–30, FSD) during the batch co-culture experiment. The error bar represents the standard deviation of four replicates.

The relative importance of phage selection pressure and bacterial competitiveness on antibiotic resistance at two stages was analyzed using the R package “relaimpo.” As shown in Fig. 7, the relative importance of phage selection pressure and bacterial competitiveness on antibiotic resistance was significantly different at two stages of coevolution (gentamycin: χ2 *df = 2* = 10.45, *P* = 0.0045; ampicillin: χ2 *df = 2* = 12.78, *P* = 0.0017; amoxicillin: χ2 *df = 2* = 10.43, *P* = 0.0053). In the ARD stage (transfer 2–10), changes in antibiotic resistance were dominated by phage selection pressure (relative importance of phage selection pressure: ≥55.47%). Whereas in the FSD stage (transfer 12–30), the effect of phage selection pressure was weakened and the effect of bacterial competitiveness was enhanced (relative importance of phage selection pressure: ≤48.83%). In contrast, the relative importance of bacterial competitiveness increased by 20.95%–22.13% in the FSD stage compared with the ARD stage (Fig. 7). Together, these results show that changes in antibiotic resistance in the ARD stage were mainly affected by phage selection pressure. In the FSD stage of coevolution, the phages tend to impact antibiotic resistance qualitatively through a direct effect on bacterial competitiveness and an indirect effect through a trade-off on bacterial competitiveness.

### Molecular evolution of *S. anatum*

**Figure 8:**
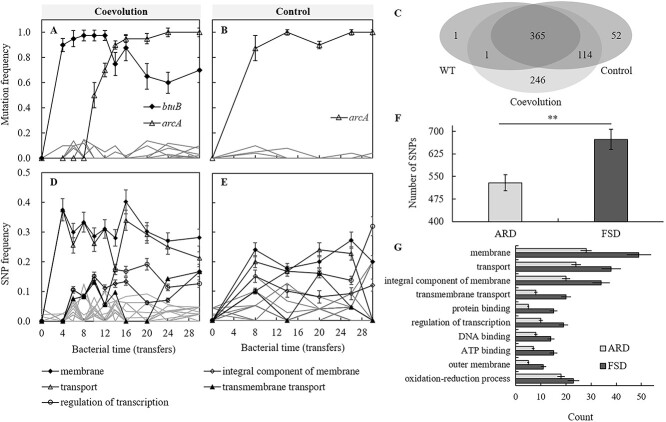
Genetic variation analysis of *S. anatum* from different transfer times during the batch co-culture experiment. (A, B) Frequency trajectories of mutant genes in bacteria isolated from coevolution and control treatments, respectively. High-frequency mutant genes were colored black. (C) Venn diagram of polymorphism sites in the genomes of bacteria isolated from coevolution treatment with phage and control without phage, respectively. (D, E) Frequency trajectories of genes with genetic polymorphism sites in bacteria isolated from coevolution and control treatments, respectively. (F) The number of polymorphism sites in the genomes of bacteria isolated form coevolution at two stages (ARD and FSD). (G) The top 10 of GO functional classification of genes with polymorphism sites in bacteria isolated from the ARD stage and the FSD stage during coevolution, respectively. The error bar represents standard deviation of four replicates.

To understand how bacteria develop mutations that lead to the trade-off described above under phage selective pressure, the phage-resistant mutants from different transfer times were re-sequenced and mapped against the WT 2089b reference genome to find the variable site. The results of genomic variation in evolved bacteria from different transfer times are shown in Fig. 8. Compared with control without phages, bacteria coevolved with phages had mutations in *btuB*, which encodes the outer membrane protein BtuB (Fig. 8A and B and Table S5). This suggests that phage JNwz02 may use BtuB as a receptor to infect the host. The previous study showed that JNwz02 had 96.57% identity with phage EPS7 and that the RBP regions of both phages are highly similar, with EPS7 using BtuB as the host receptor [28].

In addition, there were many polymorphism sites in the genome of bacteria, although the genome was extracted from bacteria growing in a single colony. The bacteria coevolved with phages had more unique polymorphism sites than the control without phages (246 vs. 52) (Fig. 8C). This suggests that phage selective pressure significantly accelerates the molecular evolution of bacteria. Based on the polymorphism sites in functional genes, 78 clusters were identified by GO enrichment analysis, and the major clusters are shown in Fig. 8D and E. The results indicate that most genes with polymorphism sites were annotated as membrane proteins (Fig. 8D and Table S6), and the antibiotic-sensitive phenotype of evolved bacteria may be explained by alterations in the cell envelope. Moreover, bacteria in the ARD stage (transfer 2–10) had fewer polymorphism sites than those in the FSD stage (transfer 12–30) during coevolution (Fig. 8F). This phenomenon may be explained by: (i) The FSD stage corresponds to more transfers than the ARD stage, resulting in a higher accumulation of mutations; and (ii) Compared with the ARD stage, the phage selective pressure in the FSD is weakened, and bacteria have more choices to develop other mutations. In addition, compared with the ARD stage, the polymorphism sites occurring by bacteria in the FSD stage are majorly focused on membrane composition and transport (Fig. 8G). This may be attributed to the fact that bacteria mainly focus on resisting the strong selection pressure of phages in the ARD stage, while in the FSD stage, bacteria further mutate genes related to membrane and transport functions to improve their competitiveness for resources due to the weakening of phage selection pressure, but this also leads to a further reduction of antibiotic resistance. Together, these results indicate that the evolution of bacteria is significantly affected by phages, and the strength of phage selective pressure is one of the important factors affecting bacterial evolution during a coevolutionary experiment.

### Evidence of BtuB as a receptor for JNwz02

**Figure 9:**
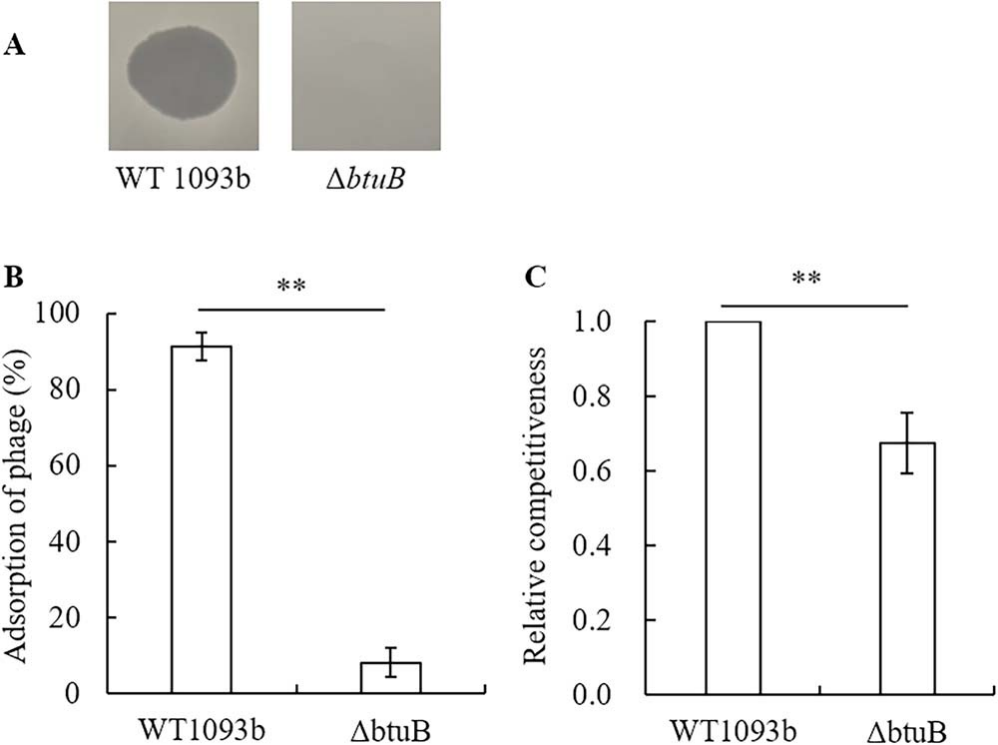
Phage adsorption rate and bacterial competitiveness of *salmonella* 1093b and Δ*btuB* strains. (A) Spot test of phages at high titers (10^9^ PFU/ml) when plated on WT 1093b and Δ*btuB*. (B) Adsorption assays of phages for WT 1093b and Δ*btuB*. (C) Relative competitiveness of WT 1093b and Δ*btuB.* The error bar represents standard deviation of three independent experiments.

We constructed a *btuB* gene deletion mutant of *Salmonella* 1093b, which is one of the hosts of JNwz02 [28], and tested the sensitivity of the mutant (Δ*btuB*) to JNwz02. Phage JNwz02 formed a clear plaque when spotted on the WT 1093b strain, while JNwz02 had no lytic activity on the Δ*btuB* (Fig. 9A). The results of the adsorption rate assay showed that the Δ*btuB* significantly reduced the adsorption rate of JNwz02 compared with WT 1093b (8.17% vs. 91.22%, *P* < 0.01, Fig. 9B). These results indicate that the outer membrane protein BtuB is a host receptor for JNwz02. In addition, the results of the competition assay showed that the competitiveness of Δ*btuB* was significantly lower than that of WT 1093b (*P* = 0.0022, Fig. 9C). Compared with WT 1093b, no change in the antibiotic resistance of Δ*btuB* was observed (data not shown). Together, these results indicate that JNwz02 utilizes BtuB as a receptor, and BtuB-mediated phage resistance leads to a decrease in bacterial competitiveness. In addition, the trade-off between phage and antibiotic resistance in bacteria is not directly affected by the mutation of *btuB* but may be indirectly affected by another mechanism, such as the change of outer membrane structure. The specific trade-off mechanism of phage and antibiotic resistance mediated by BtuB needs further study.

## Discussion

### Antagonistic coevolution between *S. anatum* and phages

The present study revealed that *Salmonella* 2089b and phage JNwz02 stably coexisted and co-evolved during the batch co-culture experiment (Figs 2 and 3), and that coevolutionary dynamics shifted from the ARD in the initial stages (transfers 2–10, Fig. 3A) to the FSD in the later stages (transfers 12–30, Fig. 3B). Kortright *et al.* [23] found that in the three replicate coevolving communities of *P. aeruginosa* and phage OMKO1, one community was undergoing ARD, whereas the other two showed evidence for FSD. More studies have shown that the coevolutionary dynamics of bacteria and phages in short-term interactions (such as <12 transfers) are usually demonstrated by ARD [22, 36, 46], while in long-term interactions (such as more than 30 transfers), it will give way from ARD to FSD [22]. One explanation for this phenomenon is that ARD is most likely not maintained long-term due to mutational constraints or fitness costs associated with defense and counter-defense, with coevolution either ending in phage extinction or giving way to FSD [22]. Moreover, the evolution of bacteria was more dominant than that of phages during the coevolutionary experiment, showing that the density of bacteria was 100 times that of phages (Fig. 2) and the phage resistance of bacteria was higher than the infectivity of phages (Figs 3 and 4A). Previous studies have similarly revealed that the coevolution system is usually ruled by bacterial resistance evolution [7, 31, 34, 35]. Potential reasons for these results may be that bacteria have more evolutionary potential than phages [47]. Bacteria evolve phage resistance primarily through mutations such as loss or alteration of phage receptors, whereas phages must acquire the ability to bind and infect a resistant host by altering their receptor-binding proteins (RBPs). The former type of mutation should outnumber the latter, as larger genomes may provide bacteria with a greater evolutionary potential than that of phages due to their more flexible array of targets [34, 47].

### The effects of competition and community in the trade-off of bacterial evolution

Coevolution could result in the emergence of a phage-resistant strain with an evolutionary cost demonstrated by a diminished competitive ability (Fig. 5). In fact, resistance-associated fitness costs are ubiquitous, as the mechanism of resistance to phage infection usually involves modifications or losses of receptors, and resistance mutants may have reduced metabolic efficiency or other vital life characteristics [13, 47]. Bacteria may not evolve mutants that are completely resistant to phages owing to a number of restrictions, such as limited resources and fitness costs. For example, bacteria from transfer 8 had the highest phage resistance (Fig. 4A), accompanied by the lowest competitiveness (Fig. 5A). It is noteworthy that long-term coevolution could lead to the emergence of a highly phage-resistant strain with no evolutionary cost, which is reflected in the recovery of bacterial competitiveness in the FSD stage (Fig. 5A). The potential explanation for this phenomenon is that, in the ARD stage of coevolution, bacterial populations were dominated by mutants with higher phage resistance but lower competitiveness, due to the intense selective pressure of phages; whereas in the FSD stage, because the selection pressure of phages is slowed down, the strains with higher competitiveness became the dominant population due to the higher efficiency of resource uptake and reproduction [27, 46]. Previous research has also revealed that both tetracycline-sensitive (*tol*C) and colistin-sensitive (LPS-related) *E. coli* mutants readily emerged, with the latter dominating in number. However, after 10 days of coevolution with phage U136B, LPS-related mutants became relatively rare due to the disparity in fitness between *tol*C and LPS-related mutations [5]. Collectively, competition complicates the coevolution between bacteria and phages, and the community structure of bacteria is shaped by the combined effects of phages and competition within populations.

### The effects of phage resistance mutations on antibiotic resistance

Bacterial resistance to three antibiotics (amoxicillin, ampicillin, and gentamycin) was significantly influenced by phages during coevolution (Fig. 6 and Table 2). In addition, the effect of phage selection pressure on the trade-off between antibiotic and phage resistance was more pronounced at the ARD stage than at the FSD stage (Fig. 7). This result indicates that coevolutionary dynamics will affect the evolutionary trade-off between phage and antibiotic resistance. Similar studies have shown that during the coevolution of *P. aeruginosa* and phage OMKO1, the trade-off between phage and antibiotic resistance occurs under ARD but not under FSD [23]. Previous studies have demonstrated that phages can drive the trade-off between phage resistance and antibiotic resistance, which is frequently reported to be related to multidrug efflux pumps [5, 8]. Gentamicin is an aminoglycoside antibiotic that kills bacteria by inhibiting protein synthesis via binding to the 16S rRNA and disrupting the integrity of the cell membrane [48]. Amoxicillin is a p-hydroxyl homolog of ampicillin; both of them kill bacteria by inhibiting the synthesis of cell walls. The antibacterial mechanism of these three antibiotics is related to the composition and structure of the membrane, and they are easy to penetrate the cell membrane [48, 49]. Considering that the bacterial resistance to three antibiotics was affected by both phage selective pressure and bacterial competitiveness (Table 2 and Fig. 6). Therefore, a more general trade-off mechanism may be that: phage JNwz02 uses a membrane component of *S. anatum* 2089b as the receptor, which is also related to nutrient uptake and metabolism. Changes in the components and structure of the membrane lead to increased membrane permeability, which in turn leads to more susceptibility to these three antibiotics. For example, the trade-off driven by phage OMKO1 might be affected by phage resistance evolution through truncated LPS, which allows bacteria to increase permeability to antibiotics [49].

### Molecular mechanisms influencing the trade-off between phage and antibiotic resistance in *S. anatum* during coevolution

The trade-off between phage resistance and antibiotic resistance is complex. Previous studies have commonly focused on the multidrug efflux pump-mediated trade-off mechanism [5], but the trade-off phenomenon proposed in this study during coevolution between *S. anatum* and phages is quite different from this mechanism. BtuB is a common phage receptor [13, 26, 50]. Our *btuB* knockout experiment also demonstrated that the outer membrane protein BtuB serves as the host receptor for phage JNwz02 (Fig. 9A and B), while establishing that BtuB is not directly associated with antibiotic resistance. Similar studies also showed that *S. enteritidis* strains with knockouts of *btuB* did not reduce resistance to gentamicin [13]. The mutation of *btuB* allows bacteria to acquire phage resistance while also reducing the uptake of Vitamin B_12_. B vitamins regulate the biomass of *Salmonella* by affecting biochemical reactions such as the metabolism of major nutrients, in which Vitamin B_12_ regulates metabolism and maintains cell structure and function by participating in biochemical reactions in the form of a prosthetic group or coenzyme [51]. Therefore, the mutation of BtuB will allow bacteria to acquire phage resistance while paying the fitness cost, which is reflected in the decrease of bacterial competitiveness (Figs 5 and 9C). Gao *et al.* [13] also demonstrated that the mutation of BtuB was associated with reduced bacterial growth. In addition, 97.5% of phage-resistant strains had *btuB* mutations, which were mainly concentrated in the ARD stage (Fig. 8A and B). Correspondingly, in the ARD stage, phage selective pressure is higher and bacterial competitiveness is lower than that in the FSD stage (Figs 4 and 5).

This study showed that BtuB-mediated phage resistance induced many mutations related to membrane components in *S. anatum* (Fig. 8D and Table S6). The alterations in membrane structure or components may lead to an increase of membrane permeability, which may be the major reason for antibiotic sensitivity. It is consistent with previous studies that Type IV pilus (T4P)-mediated adsorption deficiency-induced phage resistance and the changes in structure or composition of membranes are presumably the major cause of antibiotic sensitivity [37]. Additionally, the mutation frequency of the *btuB* gene in *S*. *anatum* decreased during the FSD stage (Fig. 8A). However, *S*. *anatum* exhibited an increased number of SNP sites (Fig. 8F) and elevated mutations associated with membrane and transport compared to the ARD stage (Fig. 8G). This phenomenon may be attributed to: (i) the FSD stage involving more transfers than the ARD stage, resulting in a higher accumulation of mutations; and (ii) in comparison with the ARD stage, the phage selective pressure in the FSD is weakened (Fig. 4B), leading *S*. *anatum* to adapt to the situation of increased growth competition within the population due to the attenuated phage selection pressure. This reason may explain the recovery of bacterial competitiveness in the FSD stage (Fig. 5A), but the corresponding reduction leads to further reduction of antibiotic resistance (Fig. 6). Together, the selection pressure of phages against *S. anatum* is different in the two coevolution patterns (ARD vs. FSD), and phage selection pressure is an important driving force for the molecular evolution of *S. anatum* in response to phage infection, thus affecting the evolution of antibiotic resistance. This discovery aligns with prior research, such as the work by Gurney *et al.* [33], have demonstrated that the mutation frequency of *P. aeruginosa* during co-evolution with phages differs under two co-evolutionary dynamics (ARD vs. FSD).

In conclusion, *S. anatum* 2089b and its virulent phage JNwz02 could undergo antagonistic coevolution, and coevolutionary arms races give way to fluctuating selection. The selection of phages causes a compromise between phage resistance and competitiveness of bacteria. Bacteria decreased their resistance to amoxicillin, ampicillin, and gentamycin during phage-driven coevolution. Compared to the FSD stage, phage selection pressure had a more significant effect on the trade-off between phage and antibiotic resistance in the ARD stage. BtuB is the host receptor for phage JNwz02, and BtuB-mediated phage resistance induced many mutations related to membrane components in bacteria, which may be the major reason for the sensitivity of phage-resistant mutants to specific antibiotics. Overall, the coevolutionary mechanism proposed in this study may be a more general trade-off mechanism between phage resistance and antibiotic resistance in bacteria. Future research will investigate the relationship between SNPs associated with membrane components in phage-resistant mutants and their antibiotic susceptibility.

## Supplementary Material

Supplementary_Information-final-20240318_ycae039

## Data Availability

Sequence data are available on NCBI (Accession number: CP118633.1, MZ570151.1). All data generated or analyzed during this study are included in this published article and its supplementary information files.
