# Supplementary material for: Phage-driven coevolution reveals trade-off between antibiotic and phage resistance in Salmonella anatum
Source: ISME Commun. 2024 Mar 22;4(1):ycae039. doi: 10.1093/ismeco/ycae039 (PMC11014889; doi:10.1093/ismeco/ycae039)
Supplement: Supplementary_Information-final-20240318_ycae039 [file supplementary_information-final-20240318_ycae039.docx]

**Phage-driven coevolution reveals trade-off between antibiotic and phage resistance in *Salmonella anatum***

Yuanyang Zhao ^ab^, Mei Shu ^b^, Ling Zhang ^a^, Chan Zhong ^b^, Ningbo Liao ^b†^, Guoping Wu ^b††^

^a^ College of Animal Science & Technology, Jiangxi Agricultural University, Nanchang, 330045, PR China

^b^ College of Food Science & Engineering, Jiangxi Agricultural University, Nanchang, 330045, PR China

**Supplementary Information**

**Table S1.** Results of analysis of deviance for the generalized linear model (quasibinomial distribution; link function=logit) of the infection probability.

| **source** | **df** | **χ^2^** | ***p*** |
| --- | --- | --- | --- |
| timeShift | 2 | 256.92 | <0.001*** |
| stage | 1 | 43.23 | <0.001*** |
| timeShift:stage | 2 | 1274.12 | <0.001*** |

**Table S2.** Results of analysis of deviance for the generalized linear model (quasibinomial distribution; link function=logit) of the bacterial resistance to phage over time during coevolutionary experiment.

| **source** | **df** | **deviance** | ***F*** | ***P*** |
| --- | --- | --- | --- | --- |
| time | 1 | 0.30486 | 9.7927 | 0.010703 * |
| time^2 | 1 | 1.24932 | 40.1308 | 8.516e-05 *** |
| time^3 | 1 | 2.00799 | 64.5012 | 1.138e-05 *** |
| time^4 | 1 | 0.56602 | 18.1818 | 0.001653 ** |

**Table S3.** Results of analysis of deviance for the linear model of relative competitiveness was fitted with transfer time as a continuous variable.

| **source** | **df** | **variance** | ***F*** | ***P*** |
| --- | --- | --- | --- | --- |
| time | 1 | 0.187812 | 35.5147 | 0.0001395 *** |
| time^2 | 1 | 0.006857 | 1.2967 | 0. 2813592 |
| time^3 | 1 | 0.221726 | 41.9276 | 7.116e-05 *** |
| time^4 | 1 | 0.142414 | 26.9300 | 0.0004075 *** |

**Table S4.** Results of analysis of deviance for the generalized linear model (quasibinomial distribution; link function=logit) of antibiotic resistance was fitted with transfer time as a continuous variable.

| **Antibiotic** |  | | **Coefficients** | | | |  |
| --- | --- | --- | --- | --- | --- | --- | --- |
|  | **Source** | **df** | | **Deviance** | ***F*** | ***p*** | |
| Ciprofloxacin | time | 1 | | 1.04896 | 16.9881 | 0.0003402 *** | |
|  | treat | 1 | | 0.02239 | 0.3626 | 0.5522756 | |
|  | time ×treat | 1 | | 0.01880 | 0.3045 | 0.5857694 | |
| Tetracycline | time | 1 | | 0.000534 | 0.0095 | 0.9232 | |
|  | treat | 1 | | 0.139633 | 2.4765 | 0.1276 | |
|  | time ×treat | 1 | | 0.023870 | 0.4234 | 0.5210 | |
| Neomycin | time | 1 | | 0.118211 | 3.7833 | 0.06266 | |
|  | treat | 1 | | 0.191952 | 6.1433 | 0.02000 * | |
|  | time ×treat | 1 | | 0.043514 | 1.3926 | 0.24864 | |
| Florfenicol | time | 1 | | 17.9993 | 208.9996 | 6.09e-14 *** | |
|  | treat | 1 | | 0.0269 | 0.3127 | 0.5808 | |
|  | time ×treat | 1 | | 0.1308 | 1.5187 | 0.2288 | |

**Table S5.** Mutants of *S. anatum* from different transfer times during the batch co-culture experiment.

| **Gene** | **Product** |
| --- | --- |
| *btuB* | TonB-dependent vitamin B12 receptor |
| *arcA* | two-component system response regulator |
| *alkB* | DNA oxidative demethylase AlkB |
| *dnaK* | molecular chaperone DnaK |
| *fliF* | flagellar basal-body MS-ring/collar protein FliF |
| *galR* | LacI family DNA-binding transcriptional regulator |
| *hsdR* | type I restriction-modification system endonuclease |
| *hslU* | HslU--HslV peptidase ATPase subunit |
| *mrcA* | peptidoglycan glycosyltransferase |
| *msrQ* | protein-methionine-sulfoxide reductase heme-binding subunit |
| *PWO88_08340* | Tn3-like element TnAs3 family transposase |
| *PWO88_12055* | OBAP family protein |
| *PWO88_19135* | MFS transporter |
| *smvA* | methyl viologen efflux MFS transporter |

**Table S6.** The top 5 classifications of genes with polymorphism sites in bacteria from coevolution treatment.

| **Function Classification** | **No. of genes** | **Gene name** |
| --- | --- | --- |
| membrane | 20 | *ompC*, *ccmB*, *oqxB*, *ansP*, *chbC*, *PWO88_00650*, *PWO88_17045*, *PWO88_17285*, *pheP*, *foxA*, *PWO88_18345*, *PWO88_20835*, *PWO88_21195*, *malF*, *btuB*, *fliF*, *ssaL*, *lpp, waaB, shdA* |
| integral component of membrane | 9 | *eptB*, *PWO88_07315*, *PWO88_07465*, *smvA*, *ybjM*, *tcuC*, *PWO88_17245*, *PWO88_20835*, *proP* |
| transport | 9 | *ompC*, *oqxB*, *ansP*, *potG*, *pheP*, *foxA*, *PWO88_17285, PWO88_18345*, *malF*, *btuB* |
| regulation of transcription | 8 | *PWO88_01645*, *PWO88_06285*, *ada*, *PWO88_08470*, *PWO88_12085*, *PWO88_12325*, *arcA*, *PWO88_21385* |
| transmembrane transport | 8 | *PWO88_07315*, *ansP*, *smvA*, *tcuC*, *PWO88_17045*, *pheP*, *PWO88_17245*, *proP* |


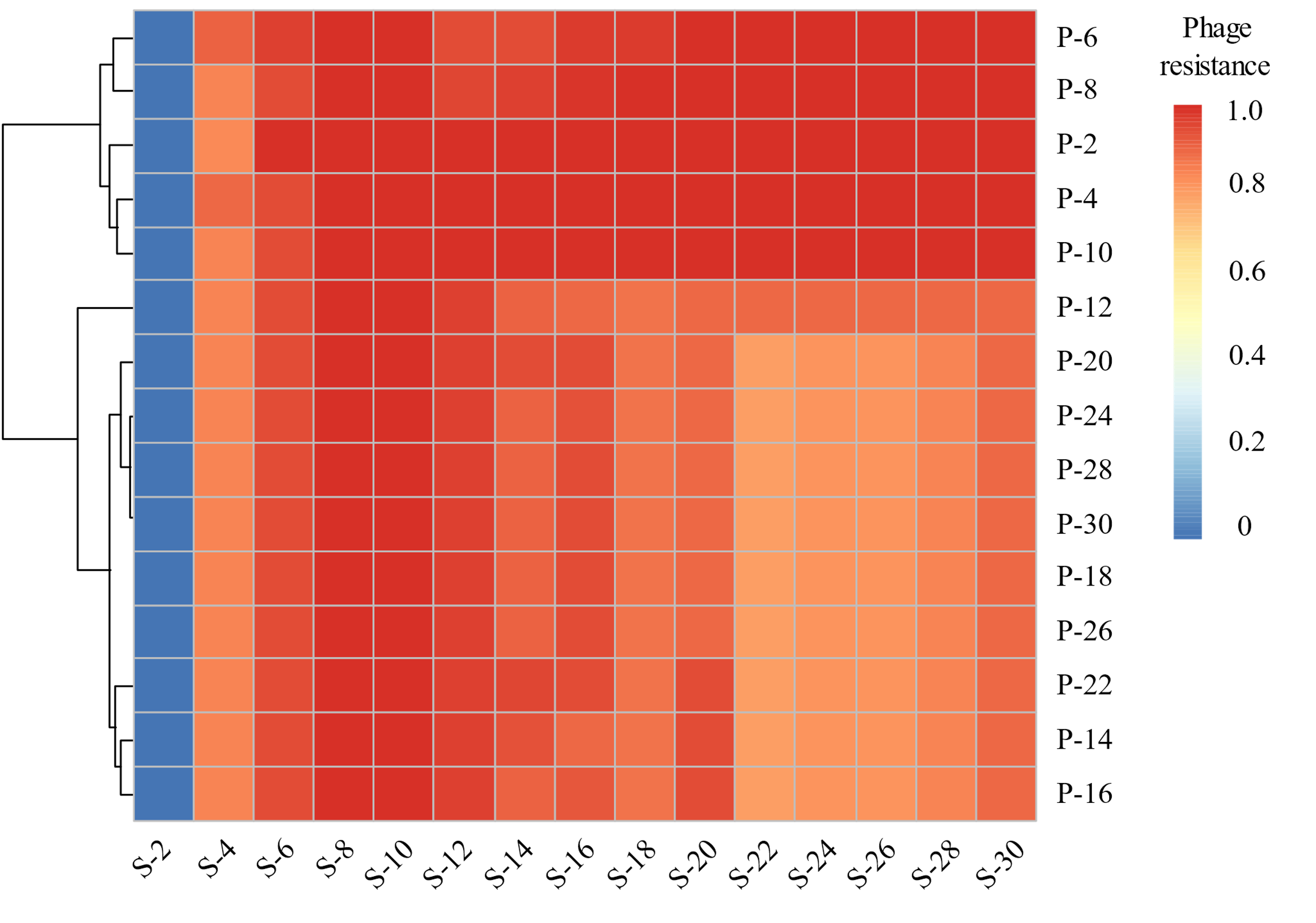


Fig. S1. The cross-infection matrices of the bacteria-phage combinations from every second transfer within the 30 transfers. The horizontal and vertical axis indicate phages and bacteria isolated from transfer 2 - 30, respectively. For example, "P-2" represents phages isolated from transfer 2.


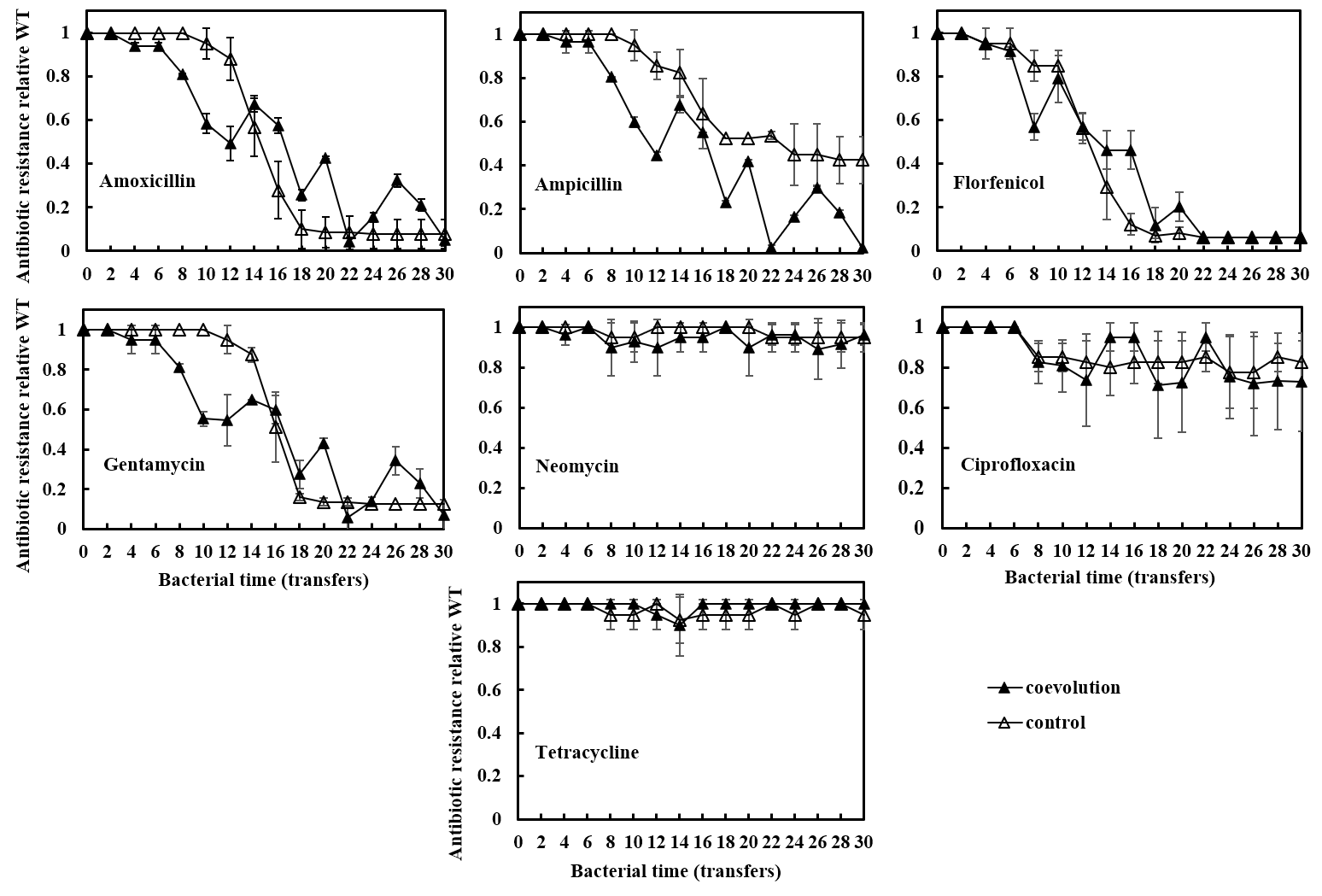


**Fig. S2.** Changes in the resistance of *S*. *anatum* to seven antibiotics over time during coevolutionary experiment.


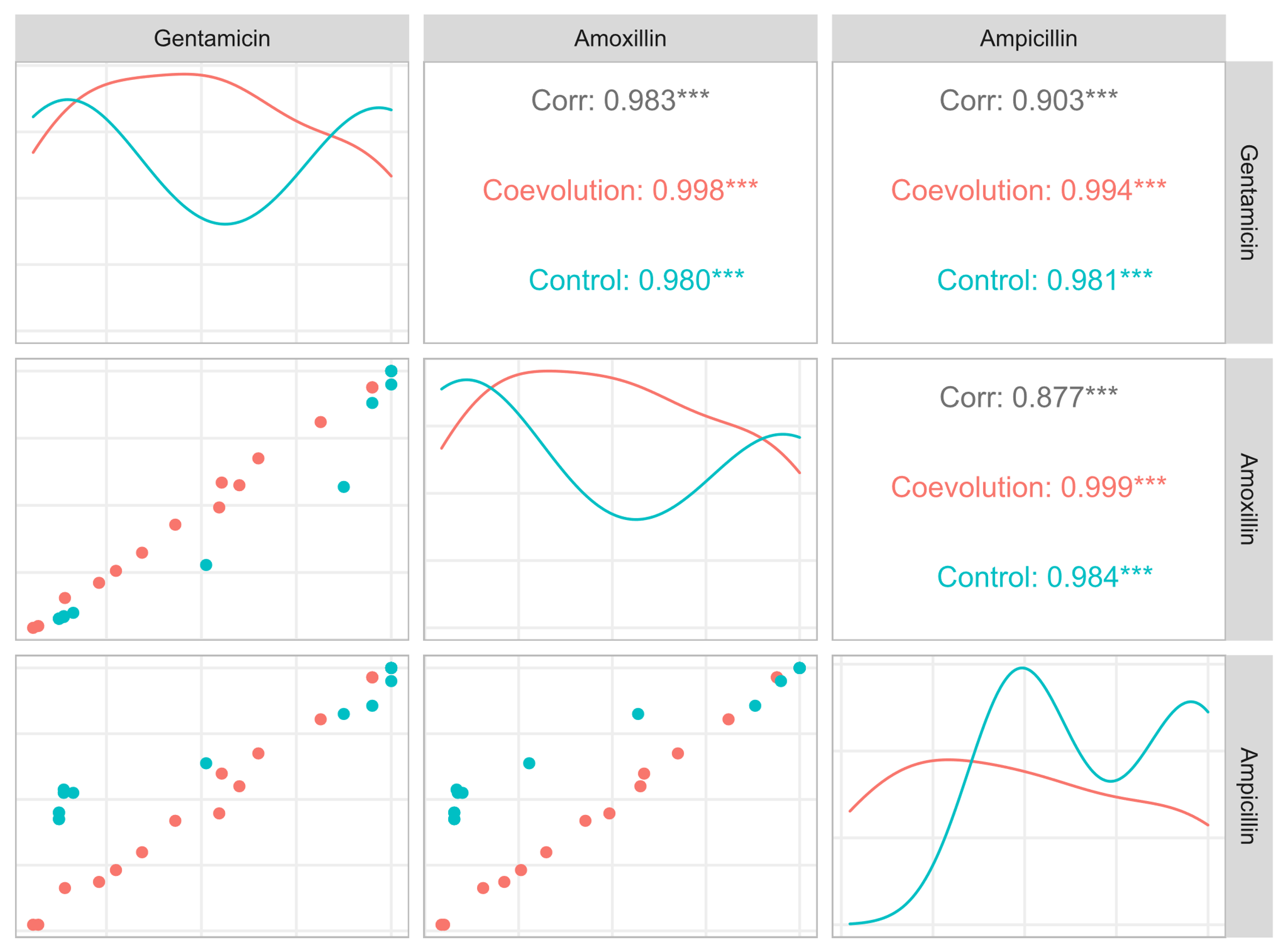


**Fig. S3.** Correlations among the antibiotic resistance of *S.* *anatum* to gentamicin, Amoxillin, and ampicillin over time during coevolutionary experiment

Selective pressure of phages ($\frac{dq}{dt})$

Gentamycin resistance

Ampicillin resistance

Amoxicillin resistance

Relative competitiveness

A

B

C

Relative competitiveness

**Fig. S4.** The effect of the interaction between phage selective pressure and bacterial competitiveness on the change of antibiotic resistance during the batch co-culture experiment. Three panels represent the relationship between resistance to the three antibiotics and relative competitiveness at five values of phage selective pressure, respectively.
